# Overview of MRI findings in progressive multifocal leukoencephalopathy

**DOI:** 10.1007/s11604-025-01837-y

**Published:** 2025-07-21

**Authors:** Koichiro Mori, Mariko Kurokawa, Masafumi Harada, Kazuo Nakamichi, Hideo Arai, Masaki Takao, Yasunobu Takaki, Yoshiharu Miura

**Affiliations:** 1 Department of Radiology, Tokyo Metropolitan Cancer and Infectious Diseases Center Komagome Hospital, 3-18-22 Honkomagome, Bunkyo-Ku, Tokyo, 113-8677 Japan; 2 Department of Radiology, The University of Tokyo, Tokyo, Japan; 3 Department of Radiology, Tokushima University, Tokushima, Japan; 4 Department of Virology, National Institute of Infectious Diseases, Japan Institute for Health Security, Tokyo, Japan; 5 Department of Pathology, Tokyo Metropolitan Cancer and Infectious Diseases Center Komagome Hospital, Bunkyō, Japan; 6 Department of Laboratory Medicine, National Center Hospital, National Center of Neurology and Psychiatry, Kodaira, Japan; 7 Department of General Internal Medicine, National Center Hospital, National Center of Neurology and Psychiatry, Kodaira, Japan; 8 Department of Neurology, Tokyo Metropolitan Cancer and Infectious Diseases Center Komagome Hospital, Bunkyō, Japan

**Keywords:** Progressive multifocal leukoencephalopathy, JC virus, Demyelination, Natalizumab, MRI, Early diagnosis

## Abstract

Progressive multifocal leukoencephalopathy (PML) is a severe demyelinating disease of the central nervous system caused by JC virus (JCV) infection. PML affects patients with various underlying conditions, such as HIV/AIDS, hematological malignancies, organ transplants, autoimmune diseases, or multiple sclerosis particularly those receiving disease-modifying therapies. MRI plays a crucial role in diagnosis, demonstrating characteristic findings across multiple sequences, including T2-weighted imaging (T2WI)/fluid-attenuated inversion recovery (FLAIR), T1-weighted imaging (T1WI), diffusion-weighted imaging (DWI), and susceptibility-weighted imaging (SWI). Early stage markers first appear as a cluster of punctate high-signal areas in T2WI (the "punctate pattern") and later develop into a distribution of oval-shaped lesions of varying sizes, commonly referred to as the "milky way appearance." Lesions typically show T2WI/FLAIR hyperintensity, T1WI hypointensity, and DWI hyperintensity. Recent findings highlight the significance of SWI hypointensity as a potential early marker. The prognosis varies significantly depending on the underlying condition and timing of diagnosis, with mortality rates ranging from 20 to 90%. Early detection, particularly in asymptomatic stages, significantly improves survival rates, emphasizing the importance of regular MRI screening in high-risk patients. Diagnostic challenges include low JCV DNA levels in cerebrospinal fluid (CSF), particularly in early stages and drug-associated cases, necessitating ultrasensitive PCR testing. This review provides an overview of PML's imaging characteristics, with particular emphasis on early diagnostic features using MRI, with a detailed understanding of PML's imaging characteristics across various stages and clinical subtypes, aiming to improve patient outcomes through early detection and intervention.

## Introduction

Progressive multifocal leukoencephalopathy (PML) is a severe demyelinating disease of the central nervous system caused by JC virus (JCV) infection [1]. It affects patients with various underlying conditions, including HIV infection/AIDS, hematological malignancies, post-organ transplantation, and autoimmune diseases [2]. In recent years, the population at risk of PML has expanded with the widespread use of disease-modifying therapy (DMT), particularly for patients with multiple sclerosis (MS). While the prognosis varies significantly depending on the underlying condition [3], it is noteworthy that ultra-early diagnosis plays a crucial role in improving outcomes [4]. Magnetic resonance imaging (MRI) plays a central role in PML screening and diagnosis by demonstrating the characteristic imaging findings across various sequences [5]. Given the potential for favorable outcomes with early detection, the importance of regular MRI screening for high-risk patients has been increasingly recognized, particularly in cases of drug-associated PML [1, 4].

This article provides a comprehensive overview of PML imaging findings, with an emphasis on MRI features that are useful for early diagnosis. We detailed the characteristic imaging findings specific to each clinical type and disease stage, aiming to assist clinicians in improving patient outcomes through early detection and intervention.

## Characteristics of the JCV: causative agents of PML

PML is a central nervous system infection caused by the JCV, which was first isolated and identified in 1971 [1]. JCV typically causes primary infection during childhood and establishes asymptomatic persistent infection in the kidneys and urinary tract while remaining latent in the lymph nodes and bone marrow [1, 2, 6]. An estimated 50–80% of adults are persistently infected with JCV [3]. During latent infection, JCV maintains a stable genomic DNA sequence referred to as an archetype [7, 8]. However, primarily in the context of cellular immune deficiency, latent JCV can reactivate and replicate within the nuclei of oligodendrocytes in the central nervous system [9]. Viral progeny released through lysis of infected cells propagate extracellularly along myelin sheaths and infect surrounding oligodendrocytes, resulting in the formation of demyelinating lesions [2, 10–12]. A distinctive characteristic of JCV detected in the brain tissue and CSF of patients with PML is the presence of patient-specific diverse mutations in the non-coding regulatory region of the viral genome, including deletions and duplications, which transform the archetype form into the prototype [6, 11, 13, 14]. Mutation into the prototype increases the virus's replication capacity, enhances its infection of oligodendrocytes, and induces demyelination, thereby elevating the risk of developing PML.

## Epidemiology and underlying diseases

The recent incidence of PML has been reported as 0.11 per 100,000 person-years in France (2010–2017) [15] and 0.029 in Japan (2016–2020) [2]. Major underlying conditions include HIV infection/AIDS, hematologic malignancies, organ transplantation, autoimmune diseases, and chronic kidney disease [1, 2]. According to a recent report from Japan [2] that analyzed 288 cases, hematological diseases accounted for 27.1% (with non-Hodgkin lymphoma comprising 53.8%), autoimmune diseases for 26.0% (with systemic lupus erythematosus (SLE) comprising 45.3%), and HIV infection/AIDS for 19.8%. In addition, solid organ transplantation represented 4.2%, solid tumors 2.8%, other conditions 13.9%, and 6.3% of cases had no apparent underlying diseases.

As of 2023, warnings regarding the development of PML existed for 18 FDA-approved drugs [16]. Notably, natalizumab has been associated with 836 reported cases as of August 2020, with an overall risk of 1 in 1,000 patients [1, 17]. However, in high-risk groups—those who are JCV antibody-positive, have over 2 years of treatment history, and prior immunosuppressant use—the incidence reaches 11.1 cases per 1,000 patients [18].

## Clinical symptoms

PML follows a characteristic course with the progressive worsening of multifocal neurological symptoms. Manifestations include cognitive and behavioral abnormalities, sensory and motor deficits, ataxia, aphasia, visual disturbances, and seizures [1, 19, 20]. Initial symptoms most frequently present are cognitive impairment and neuropsychiatric symptoms, followed by motor dysfunction, visual disturbances, coordination and gait disorders, sensory disturbances, and epileptic seizures [3, 21].

## Treatment and prognosis

The key therapeutic principle is to restore JCV-specific immunity [3]. For HIV-associated PML, the introduction of antiretroviral therapy (ART) is most effective, whereas for non-HIV-associated PML, discontinuation of immunosuppressive therapy to achieve immune reconstitution is considered most beneficial [1]. Recently, immune checkpoint inhibitors have shown promising results in some cases [22]. However, a direct antiviral therapy has not been established [1].

The prognosis varies significantly depending on the underlying condition, with mortality rates ranging from 20 to 90% [3]. PML patients with hematologic malignancies have been reported to have a 90% mortality rate within 2 months [1, 23–25]. In HIV-infected patients, the median survival time before ART introduction was 6 months, with only 9% surviving beyond a year [26]. However, early introduction of ART leads to clinical stabilization or improvement in 44–83% of cases [27]. Among long-term survivors, approximately 70% often retain some neurological deficits [1, 27, 28]. In natalizumab-associated PML, shorter time to diagnosis and localized lesions are associated with better functional outcomes [4, 29].

## MRI findings of PML

PML lesions can occur in various locations, from the supratentorial to infratentorial structures. Each MRI sequence [T2-weighted imaging (T2WI)/fluid-attenuated inversion recovery (FLAIR), T1-weighted imaging (T1WI), diffusion-weighted imaging (DWI), and susceptibility-weighted imaging (SWI)] demonstrated characteristic findings, and a comprehensive evaluation of these sequences is crucial for an accurate diagnosis. The following sections describe the main imaging findings of PML.

### Supratentorial lesions

PML showed a predominance of supratentorial lesions (87.7%) compared to infratentorial lesions (27.4%) [30]. Lesions can occur simultaneously in both supratentorial and infratentorial regions. However, PML rarely affects the spinal cord [31]. In the supratentorial regions, lesions are most frequently observed in the frontal (64.1%) and parietal lobes (46.6%), although lesions can also develop in the temporal and basal ganglia regions [30]. Although multifocal lesions are characteristic of PML, single lesions can also occur [32].

**Fig. 1:**
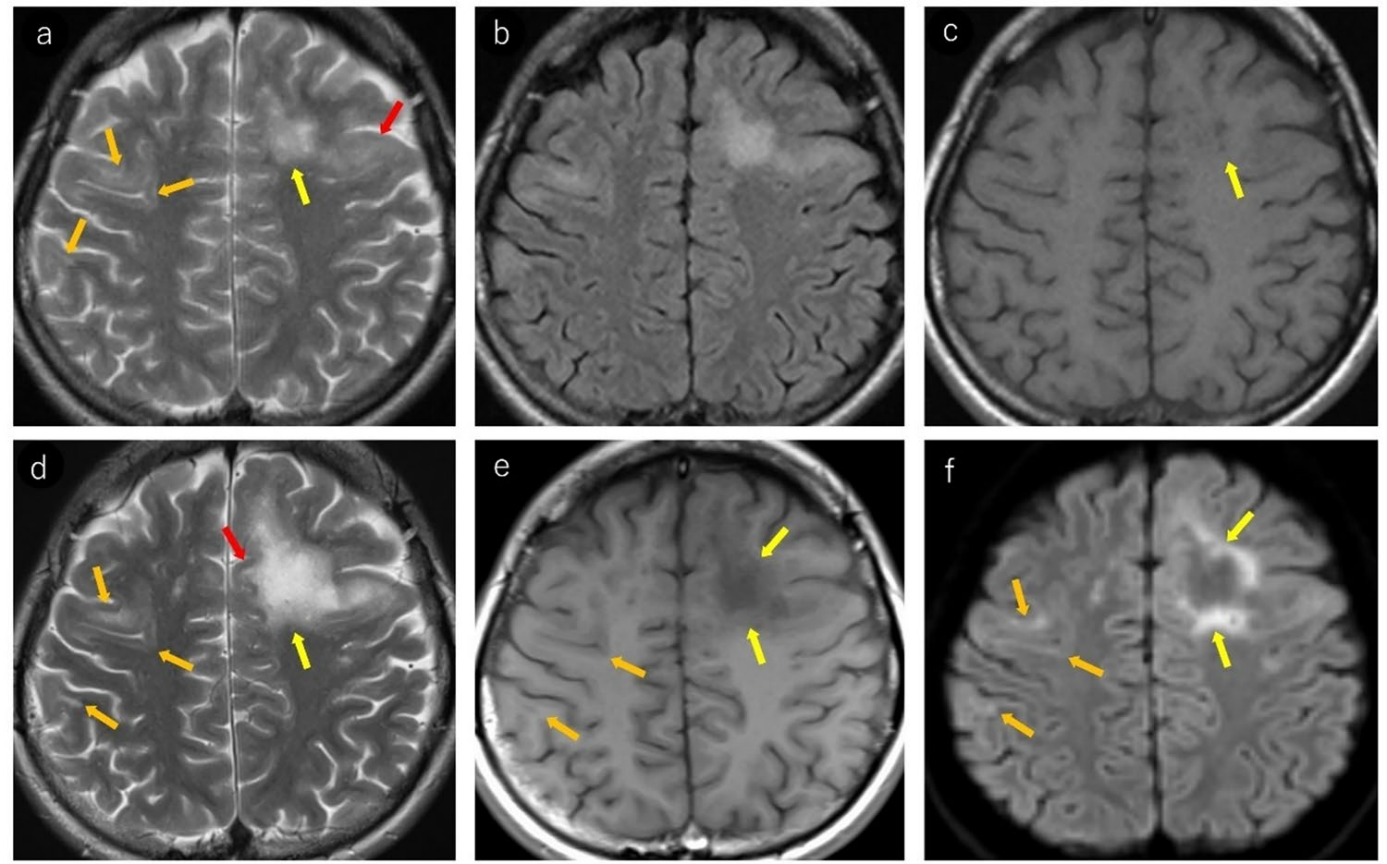
Woman in her 30 s with a known medical history of HIV infection presented with symptoms of mild paralysis in her right lower extremity and exhibited mild articulation disorder. Notably positive JCV–PCR in the cerebrospinal fluid, confirmed a diagnosis of PML. Initial MRI findings (**a–c**): **a** T2-weighted imaging reveals heterogeneous hyperintense lesions in the left frontal lobe extending from the corticomedullary junction to the subcortical white matter (yellow arrows). Subtle hyperintensity is also noted in the left frontal cortex (red arrows). Subtle linear T2 hyperintense lesions are observed at the corticomedullary junction of the right frontal and parietal lobes (orange arrows). **b** FLAIR image demonstrates more conspicuous bilateral frontal and right parietal lesions, enhancing their detectability. **c** Left frontal lesion appears as a mildly heterogeneous hypointense area on T1-weighted images (yellow arrows). Right frontal and parietal lesions were not identifiable. Follow-up MRI after a month (**d–f**): **d** linear T2 hyperintense lesions at the corticomedullary junction of the right frontal and parietal lobes show slight progression in size and conspicuity (orange arrows). The left frontal lesion expanded, exhibiting sharp margins on the cortical side (red arrows) and ill-defined borders on the white matter side (yellow arrows). **e** On T1-weighted imaging, the left frontal lesion demonstrates heterogeneous hypointensity with a central CSF-like signal intensity (yellow arrows). Corticomedullary junction lesions in the right frontal and parietal lobes also show subtle hypointensity (orange arrows). **f** Left frontal lesion exhibits a rim and core pattern on diffusion-weighted imaging, with a hyperintense periphery and a hypointense center (yellow arrows). Corticomedullary junction lesions in the right frontal and parietal lobes showed subtle linear hyperintensity (orange arrows) and were readily identifiable

On MRI, FLAIR excels in lesion detection, with lesions appearing hyperintense [5]. Lesions typically extend from subcortical regions, including U-fibers, to the subcortical white matter. T2WI is well-suited for delineating lesion borders, showing relatively sharp boundaries on the cortical side but somewhat ill-defined margins on the white matter side [33]. In addition, individual lesions may demonstrate heterogeneous signal intensities, and intralesional vacuoles are considered characteristic of PML [5, 34–37] (Fig. 1).

### Cerebral cortical and gray matter lesions

**Fig. 2:**
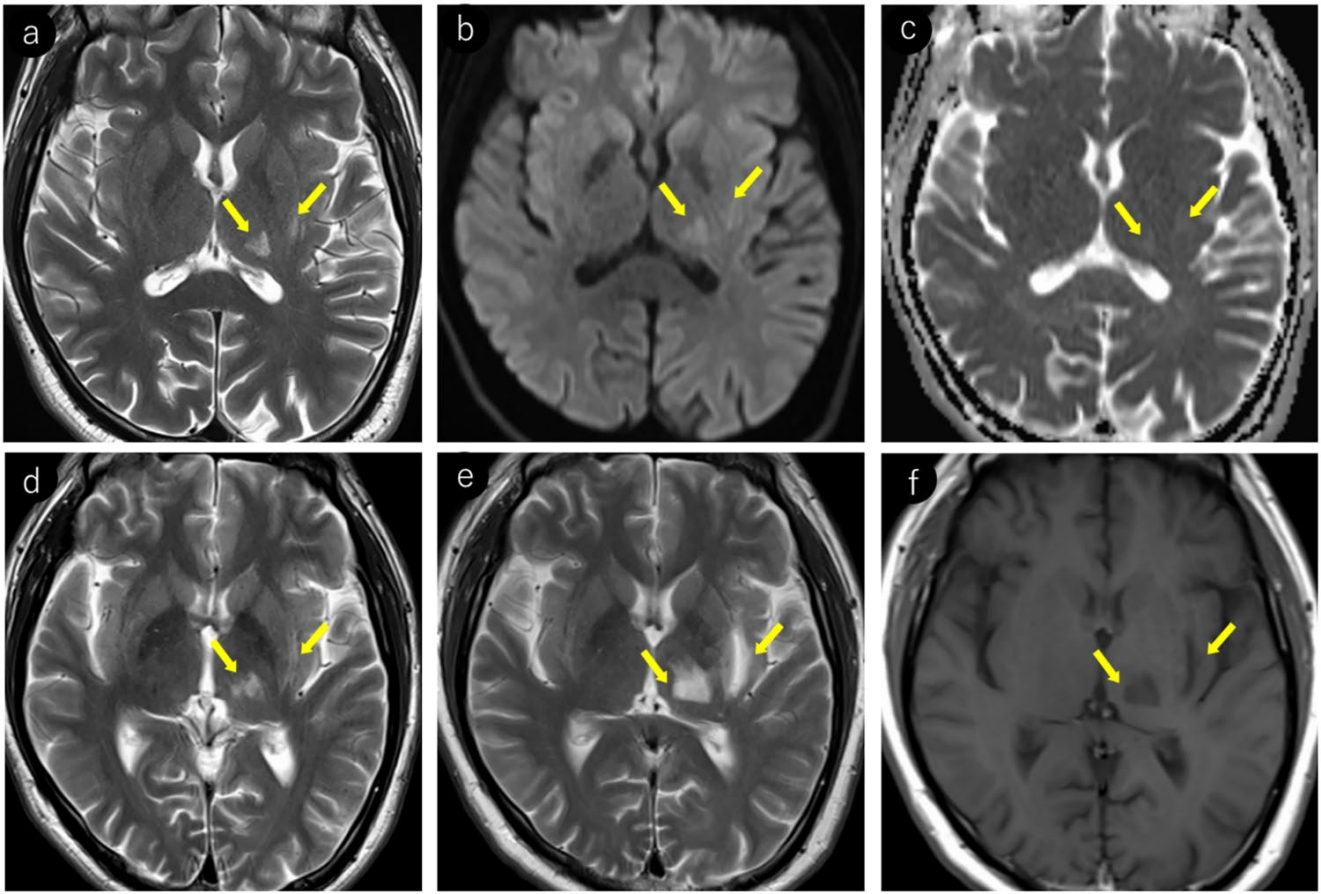
HIV-positive man in his 30 s began experiencing a sensation of heaviness in his right lower limb approximately 3 months prior, which gradually worsened. A week prior, there was a rapid loss of mobility in the right lower limb, and similar symptoms manifested in the right upper limb. Initial MRI findings (**a–c**): **a** T2-weighted image demonstrating hyperintense lesions in the left thalamus and lateral putamen (yellow arrows). **b** These lesions show mild hyperintensity on diffusion-weighted imaging (yellow arrows). **c** ADC map showing increased values without diffusion restriction (yellow arrows). Follow-up MRI findings (**d–f**): **d** 2-month follow-up imaging shows progression of the lesions with enlargement (yellow arrows). **e** Further progression with expansion of the lesions was observed at the 4-month follow-up (yellow arrows). **f** Lesions appear hypointense on T1-weighted imaging, with the lateral putaminal lesion showing a CSF-like signal intensity (yellow arrows). A cerebrospinal fluid examination confirmed the presence of the JCV, leading to a diagnosis of PML

JCV infects not only oligodendrocytes in the cerebral white matter but also neurons in the cortex and deep gray matter [38–40]. Reports indicate that gray matter lesions are present in approximately 57% of patients with PML [39]. However, cerebral cortical lesions have a low detection sensitivity on MRI [1] and appear as subtle hyperintensities on T2WI/FLAIR (Fig. 1). These lesions frequently occur near the corticomedullary junction and are often associated with subcortical white matter lesions [39]. In addition, lesions in deep gray matter structures such as the thalamus and putamen may present with a lacunar infarct-like appearance [40]. The key differentiating features included the absence of reduced apparent diffusion coefficient (ADC) values and progressive enlargement over time (Fig. 2).

### Infratentorial lesions (infratentorial PML)

**Fig. 3:**
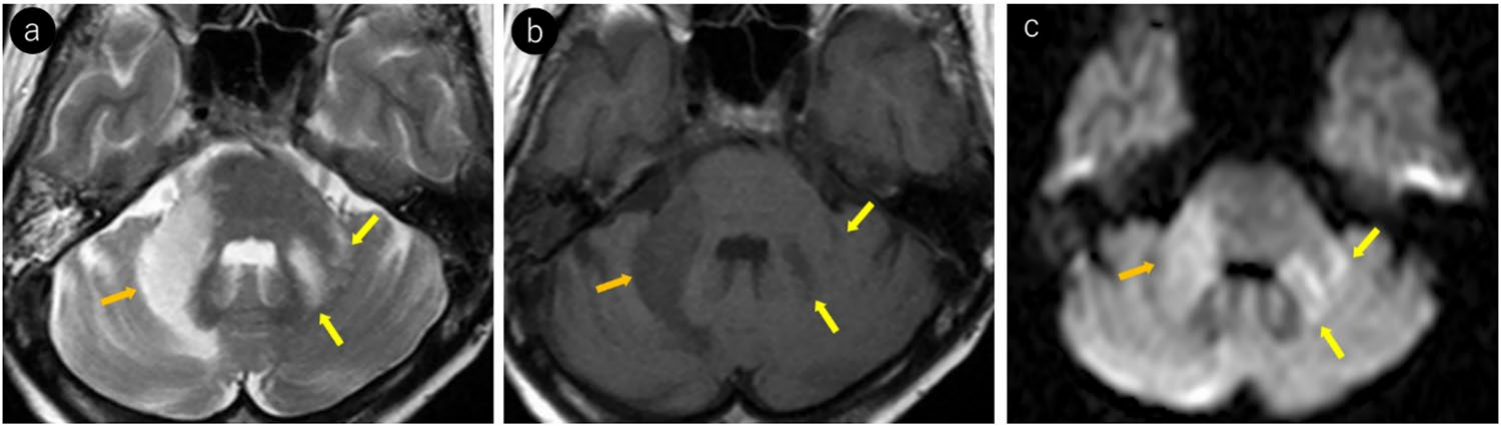
Woman in her 50 s with post-bone marrow transplantation for acute myeloid leukemia presented with progressive cerebellar ataxia. **a** T2-weighted imaging reveals hyperintense lesions around the right dentate nucleus extending to the right middle cerebellar peduncle, demonstrating a “shrimp sign” (orange arrows). Additional T2 hyperintense lesions were noted around the left dentate nucleus and left middle cerebellar peduncle (yellow arrows), showing an asymmetric distribution. **b** Lesions appear hypointense on T1-weighted images (orange and yellow arrows). **c** Most lesions demonstrate hyperintensity on diffusion-weighted imaging (orange and yellow arrows). The presence of JCV in the cerebrospinal fluid was confirmed and PML was diagnosed

PML can involve infratentorial structures, with lesions frequently occurring around the dentate nucleus of the cerebellum, middle cerebellar peduncles, and the brainstem [40, 41]. A characteristic pattern of spread along the peridentate region including the dentate nucleus has been described as the “shrimp sign” [40, 42] (Fig. 3). Lesions can be bilateral and typically present with an asymmetric distribution [42]. Although rare, brainstem-limited PML have been reported [43].

### “Punctate pattern,” “milky way appearance”

**Fig. 4:**
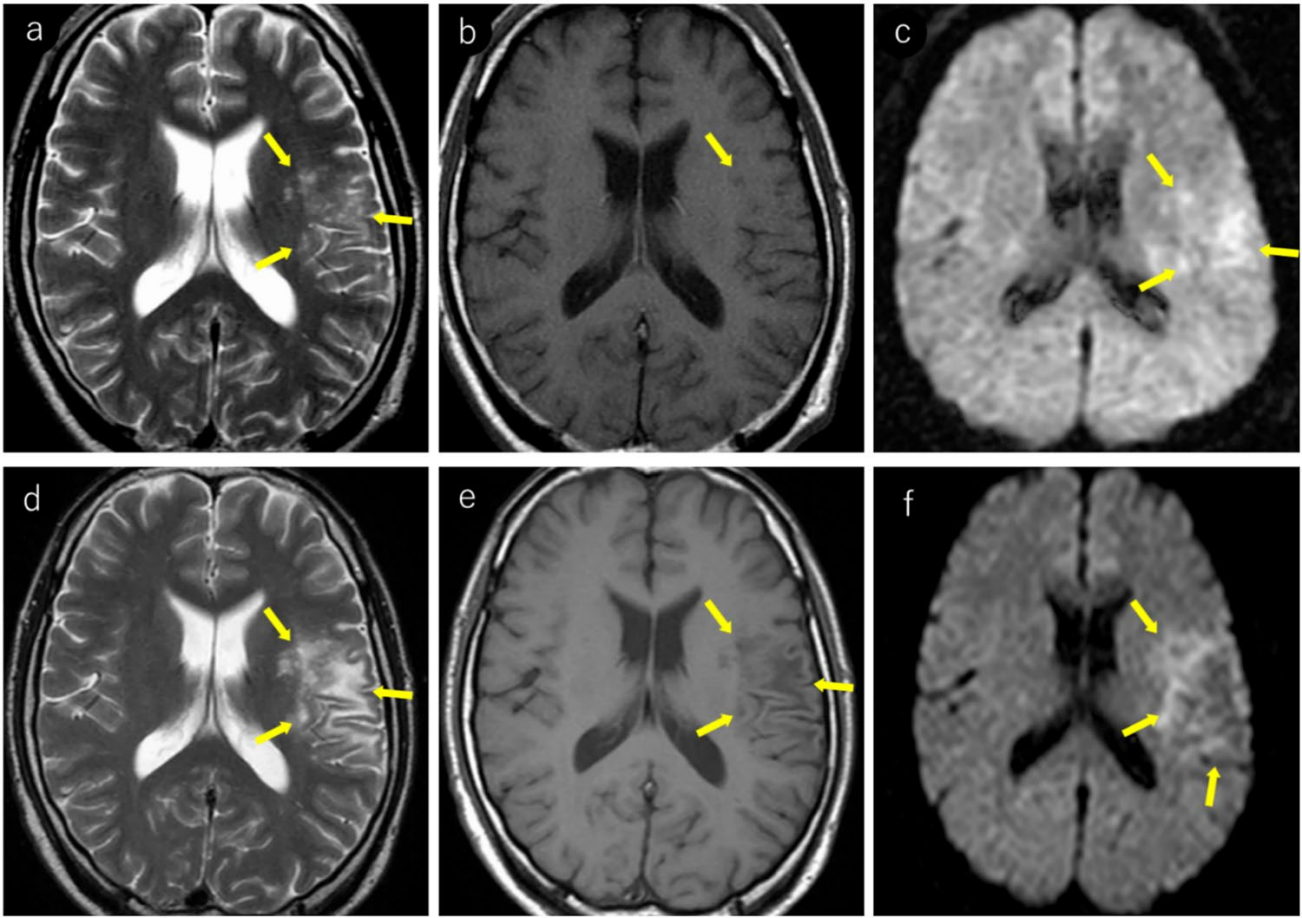
HIV-positive man in his 40 s underwent brain MRI due to weight loss and general fatigue. Initial MRI findings (**a–c**): **a** T2-weighted imaging showing punctate hyperintense lesions in the subcortical white matter, primarily affecting the left operculum and insula (yellow arrows). **b** Some lesions demonstrate subtle hypointensity on T1-weighted images (yellow arrow). **c** T2 hyperintense areas show corresponding hyperintensities on diffusion-weighted imaging (yellow arrows). PCR testing for JCV in the CSF was negative. After the onset of progressive articulation disorder and decreased muscle strength in the right upper limb, a repeat MRI was performed after 2 months. Follow-up MRI after 2 months (**d–f**): **d** T2-weighted imaging demonstrates progression with expansion of the lesions (yellow arrows). **e** Lesions appear heterogeneously hypointense on T1-weighted images (yellow arrows). **f** Diffusion-weighted image revealing predominantly peripheral hyperintensities (yellow arrows). A repeat PCR test for CSF JCV confirmed positivity, leading to the diagnosis of PML

**Fig. 5:**
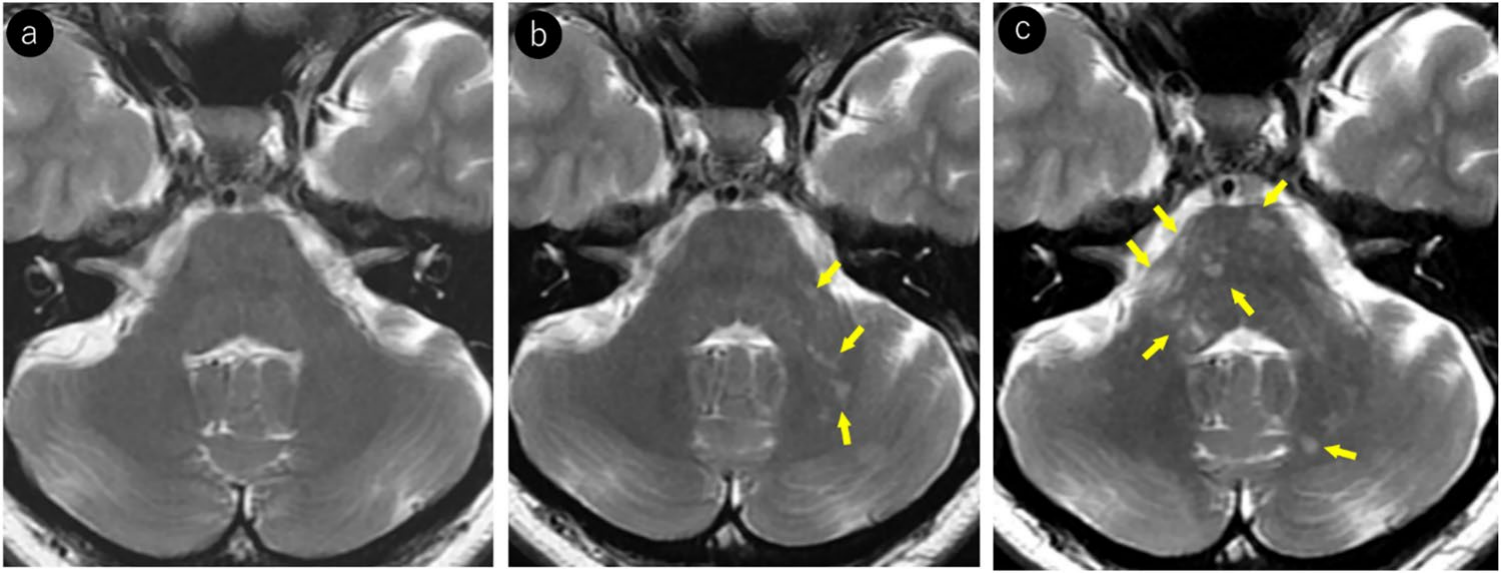
Woman in her 40 s, who had been undergoing fingolimod treatment for MS for 8 years started experiencing walking difficulties a few weeks prior to presentation. **a** MRI performed 3.5 years prior showing no abnormalities in the infratentorial region. **b** T2-weighted imaging 2 years prior demonstrating subtle punctate and small hyperintense lesions around the left dentate nucleus and left middle cerebellar peduncle (yellow arrows). These lesions were considered asymptomatic at the time as the patient exhibited no clinical symptoms. **c** At the time of symptom onset, T2-weighted imaging reveals a punctate pattern and milky way appearance in the pons, bilateral middle cerebellar peduncles (right predominant), and around the left dentate nucleus (yellow arrows). JCV was detected in cerebrospinal fluid examination and PML was diagnosed

**Fig. 6:**
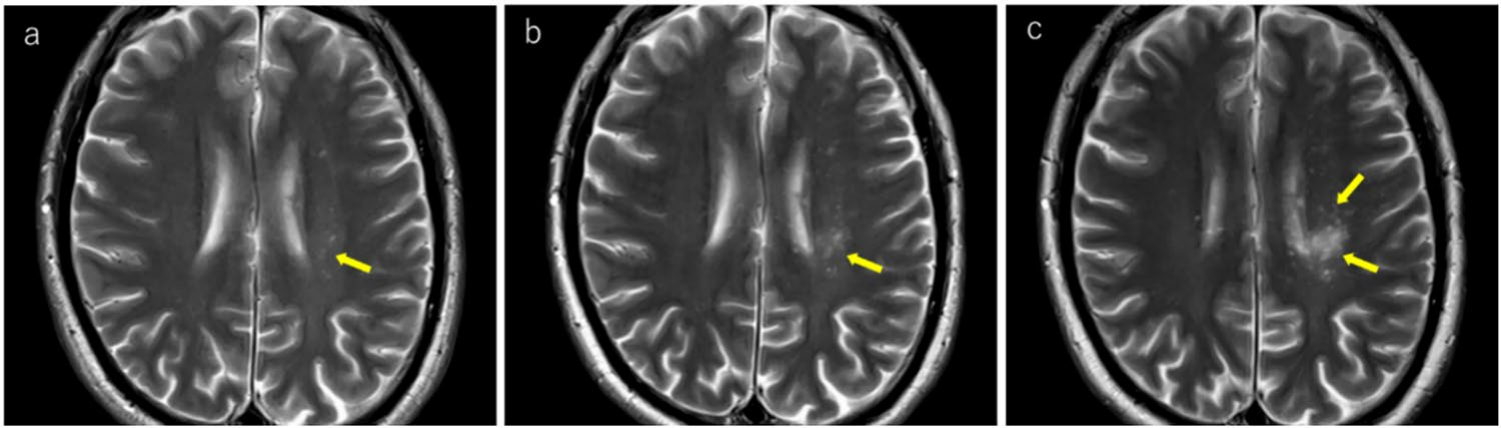
Same patient as in Fig. 2: an HIV-positive man with PML in his 30 s. **a** T2-weighted image showing the emergence of a punctate pattern in the deep white matter around the left lateral ventricle (yellow arrow). **b** One month later, the lesions show local expansion (yellow arrow). **c** After an additional month, the lesions showed further local expansion and milky way appearance (yellow arrows)

**Fig. 7:**
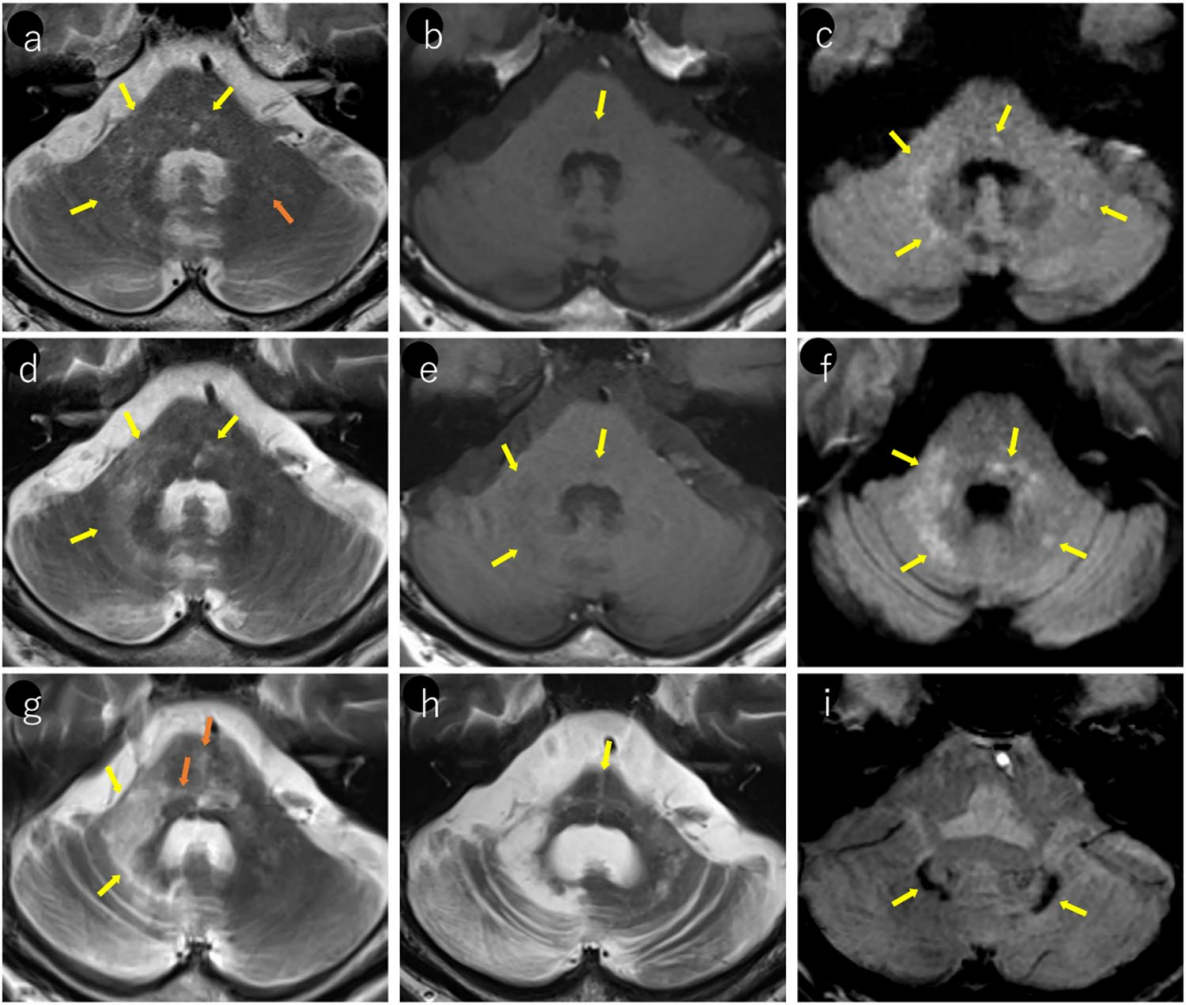
Man in his 60 s with no significant medical history presented with progressive difficulty in using chopsticks with his right hand, which began 3 months prior to presentation. Two months prior, he developed dysgraphia, followed by gradual onset of dysarthria. Initial MRI findings (**a–c**): **a** T2-weighted image showing a punctate pattern extending from the right dentate nucleus to the right middle cerebellar peduncle and pons (yellow arrows). Subtle lesions are also noted around the left dentate nucleus (orange arrow), showing an asymmetric distribution. **b** Some lesions appear mildly hypointense on T1-weighted images (yellow arrow). **c** Lesions show subtle hyperintensities on diffusion-weighted imaging (yellow arrows). One-month follow-up MRI (**d–f**): **d** lesions involving the right dentate nucleus, right middle cerebellar peduncle, and pons have expanded and demonstrated a milky way appearance (yellow arrows). **e** T1-weighted image showing a slight progression of hypointensity (yellow arrows). **f** Lesions become more conspicuous with enhanced hyperintensity on diffusion-weighted imaging (yellow arrows). JCV was detected in cerebrospinal fluid examination and PML was diagnosed. Three-month follow-up MRI (**g**): **g** T2-weighted imaging clearly demonstrates the “shrimp sign” (yellow arrows) with right cerebellar hemisphere atrophy. The pons shows a cross sign with lesion progression along the transverse pontine fibers (orange arrows). One-and-a-half-year follow-up MRI (**h–i)**: **h** marked cerebellar atrophy predominantly on the right side. The pons showed atrophy with a persistent cross-sign (yellow arrow). **i** SWI demonstrating hypointensity in the dentate nucleus adjacent to the lesions (yellow arrows)

The “punctate pattern” (Figs. 4, 5, 6, 7), characterized by clustered punctate lesions on T2WI, and the “milky way appearance” (Figs. 4, 6), showing multiple tiny lesions clustering around the main lesion, have been reported as characteristic MRI findings in asymptomatic PML and early stage PML [5, 34, 44, 45]. The punctate pattern is commonly observed near the cortico-medullary junction or subcortical white matter, but can also be seen in the deep white matter [45]. In infratentorial regions, it was frequently observed around the dentate nucleus, middle cerebellar peduncles, or pons (Fig. 7). The punctate pattern often presents as an initial finding that precedes the development of the “milky way appearance” or typical PML lesions [40, 45] (Figs. 4, 6, 7).

### T1-weighted imaging findings

PML lesions appear iso- to hypointense on T1WI and progressively become more hypointense until approaching CSF signal intensity as the disease advances (Figs. 1, 2, 3, 4). This indicates severe demyelination, tissue destruction, and minimal remyelination. However, smaller and early stage lesions, such as those showing a punctate pattern, were less likely to demonstrate T1WI hypointensity (Figs. 4, 7). In addition, heterogeneous hypointensity within the same lesion on T1WI, reflecting varying degrees of tissue destruction, is another important finding suggestive of PML [1, 4, 46].

**Fig. 8:**
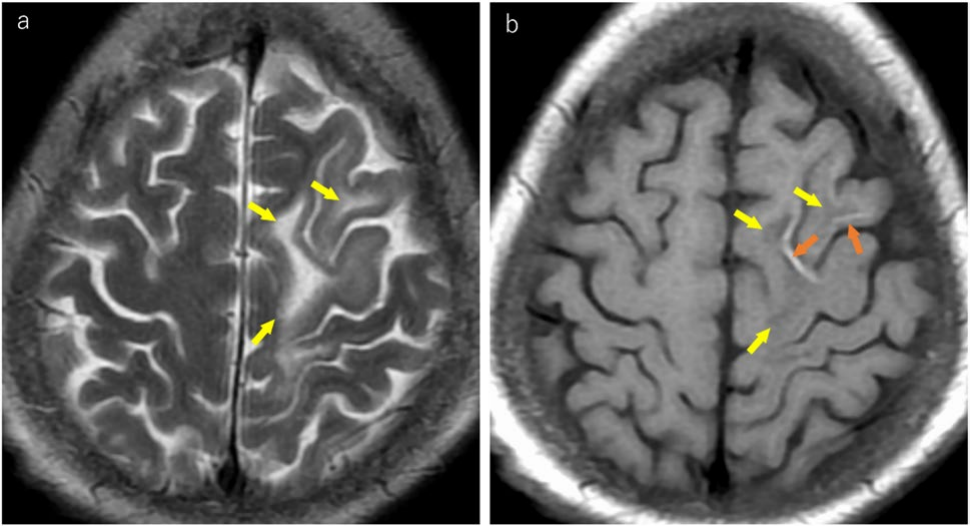
Woman in her 70 s with severe instability and anemia was diagnosed with diffuse large B-cell lymphoma (DLBCL). Subsequently, the patient underwent chemotherapy. After the treatment, the patient presented with right-sided hemiparesis. The patient also exhibited progressive articulatory disorders and decreased swallowing function. JCV was detected in the cerebrospinal fluid, confirming a diagnosis of PML. **a** T2-weighted image revealing hyperintense lesions in the subcortical white matter of the left frontal lobe (yellow arrows). **b** On T1-weighted imaging, lesions appear hypointense (yellow arrows). The adjacent cortex shows hyperintensity (orange arrows)

Furthermore, hyperintense cortical signal (HCS) has been reported as a finding, where cerebral cortex and deep gray matter adjacent to PML lesions show hyperintensity on T1WI [44, 47] (Fig. 8). While this finding is frequently observed in patients with PML following seizures or after immune reconstitution inflammatory syndrome (IRIS), it can also occur in patients with PML without seizures or IRIS [47]. Pathologically, HCS is associated with marked demyelination of the cerebral cortex and U-fibers, prominent macrophage infiltration, and severe reactive gliosis [47].

### Diffusion-weighted imaging (DWI)

The periphery of the PML lesions, characterized by viral replication and oligodendrocyte swelling, showed hyperintensity on DWI, which is considered to reflect disease activity (Figs. 1, 3, 4, 7). When lesions show hyperintensity at the periphery and hypointensity at the center on DWI, it is referred to as a rim-and-core pattern (Fig. 1). While ADC values are typically elevated, areas of restricted diffusion may be observed in the periphery of rapidly expanding lesions, which are thought to be caused by the swelling of dying oligodendrocytes and astrocytes [5, 48–51]. Early stage findings such as a punctate pattern, milky way appearance, or subtle T2WI lesions may show mild hyperintensity on DWI, making them extremely valuable for diagnosis (Figs. 4, 7) [51]. However, it is important to note that in asymptomatic patients with PML, cases with less prominent DWI hyperintensity have been reported, suggesting limited damage to oligodendrocytes and astrocytes [52].

### Susceptibility-weighted imaging (SWI) findings

**Fig. 9:**
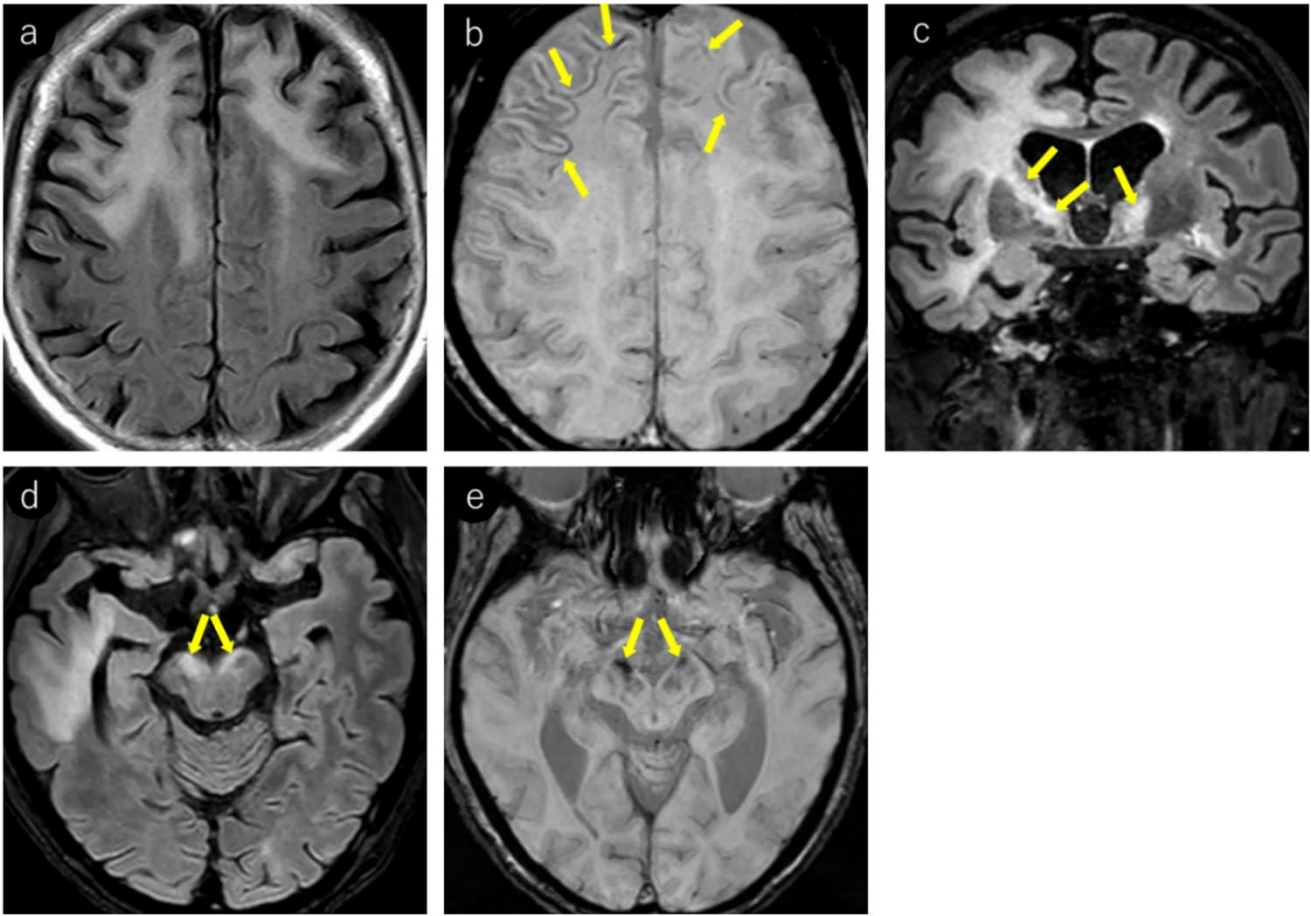
Woman in her 80 s who developed PML during chemotherapy for DLBCL. MRI findings were obtained when the patient presented with disturbed consciousness 4 months after the onset of PML. **a** Fluid-attenuated inversion recovery (FLAIR) imaging shows hyperintense lesions in the bilateral frontal lobes, predominantly on the right side, extending from the juxtacortical to the subcortical white matter. **b** Linear hypointensity is observed predominantly on the right side of the SWI in the deep cortical layers and the corticomedullary junction adjacent to the lesions (yellow arrows). **c** Coronal FLAIR images demonstrating right-predominant caudal lesion progression along the corticospinal tract (yellow arrows). **d** Bilateral hyperintense lesions are noted in the medial cerebral peduncles on FLAIR imaging, which are more prominent on the right side (yellow arrows). **e** Substantia nigra lesions show hypointensity on SWI with a right-sided predominance (yellow arrows)

**Fig. 10:**
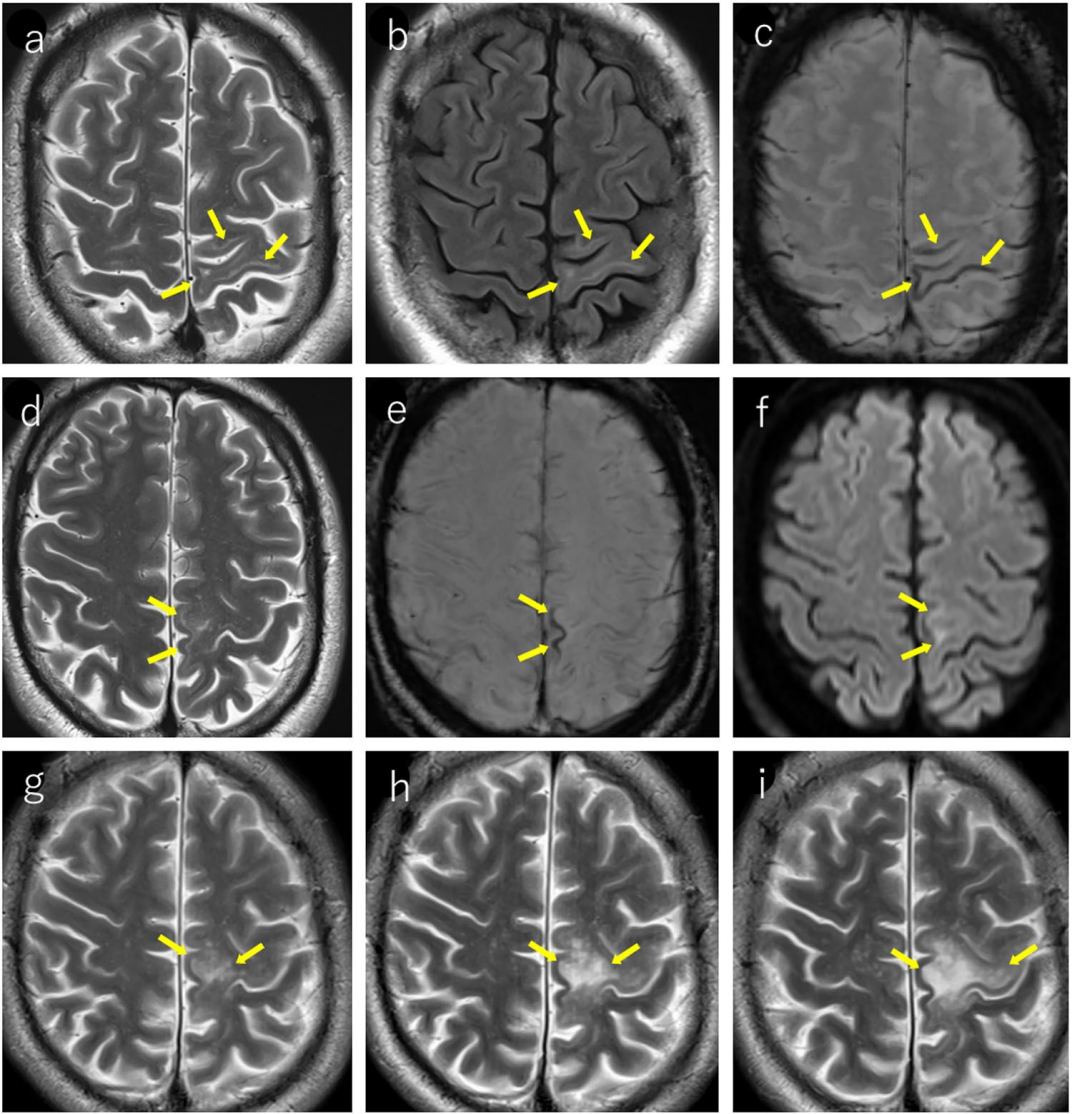
The same patient as in Fig. 2: an HIV-positive man with PML in his 30 s. Initial MRI findings (**a–f**): **a** linear T2 hyperintense lesions were observed near the corticomedullary junction, primarily in the left precentral gyrus (yellow arrows). **b** These lesions appear more conspicuous as hyperintense areas on FLAIR imaging (yellow arrows). **c** Linear hypointensity is noted on SWI in the cortex adjacent to the lesions (yellow arrows). 5-mm caudal slice findings (**d–f**): **d** linear T2 hyperintense lesions present at the cortico-medullary junction of the left precuneus (yellow arrows). **e** SWI showing linear hypointensity in the deep cortical layers and cortico-medullary junction in parts of the lesion (yellow arrows). **f** Lesions demonstrating linear hyperintensity on diffusion-weighted imaging (yellow arrows), making them readily identifiable. Follow-up findings (**g–i**): **g** 2-month follow-up showing lesion expansion into the subcortical region with a milky way appearance (yellow arrows). **h**, **i** Further progression of lesions within the subcortical white matter was observed at the and 4-month follow-up (yellow arrows)

SWI is an imaging technique that detects magnetic susceptibility changes excelling in the detection of microhemorrhages and hemosiderin deposits. Since the initial report of SWI hypointensity in U-fiber regions in PML cases [53], there has been an increasing number of reports on the clinical utility of SWI. Studies have shown that 64% of patients with PML demonstrate SWI hypointensity at the grey–white matter (cortico-medullary) junction [54]. SWI hypointense lesions can be observed in various regions, including the corticomedullary junction [54] [55] (Figs. 9, 10) and cortex adjacent to subcortical lesions [56] (Fig. 10). As the disease progresses, these lesions can extend to deep gray matter structures, such as the thalamus and basal ganglia, as demonstrated by Miyagawa et al. [53]. The hypointense signal of the dentate nucleus (Fig. 7) and substantia nigra (Fig. 9) shown on SWI may also represent important findings of PML. SWI hypointense findings can appear at various stages, from the initial diagnosis or early disease to chronic phases [54, 57].

In some cases, cortical SWI hypointensity was observed adjacent to subtle, localized T2WI/FLAIR hyperintensity in subcortical lesions or subcortical white matter [54] and reports of cortical SWI hypointensity preceding the appearance of subcortical white matter signal abnormalities [57], suggesting that SWI findings may serve as early diagnostic markers before lesion expansion. Pathologically, SWI–hypointense regions corresponded to iron deposits within macrophages, suggesting inflammatory changes [54]. The formation of SWI hypointense lesions have been attributed to blood–brain barrier (BBB) leakage along perivascular spaces [58]. In terms of prognostic significance, the SWI hypointense rim represents a marker of the end-stage neuroinflammatory process in long-term survivors [58]. The presence of this rim correlates with prolonged survival and is associated with more pronounced reactive changes at the subcortical boundary [58]. However, it should be noted that SWI findings are not present in all PML cases, and similar findings can be observed in conditions, such as cerebral infarction, encephalitis, amyotrophic lateral sclerosis, or age-related changes et al. [59], necessitating careful interpretation.

## Correlation between pathological progression pattern and MRI findings of PML

Ono et al. [40] provided a detailed report on the correlation between pathological progression patterns of PML and MRI findings. According to their report, the progression of PML can be divided into three stages.

- First step: “Initiation”—The stage where small demyelinating lesions (initial infectious demyelinating foci) are formed.
- Second step: “Expansion” and “Extension”—The stage where viral replication occurs, and demyelinating lesions expand and progress into surrounding tissues.
- Third step: “Fusion”—The stage where multiple demyelinating lesions merge and axonal destruction occurs.

### Correlation with MRI findings

**Fig. 11:**
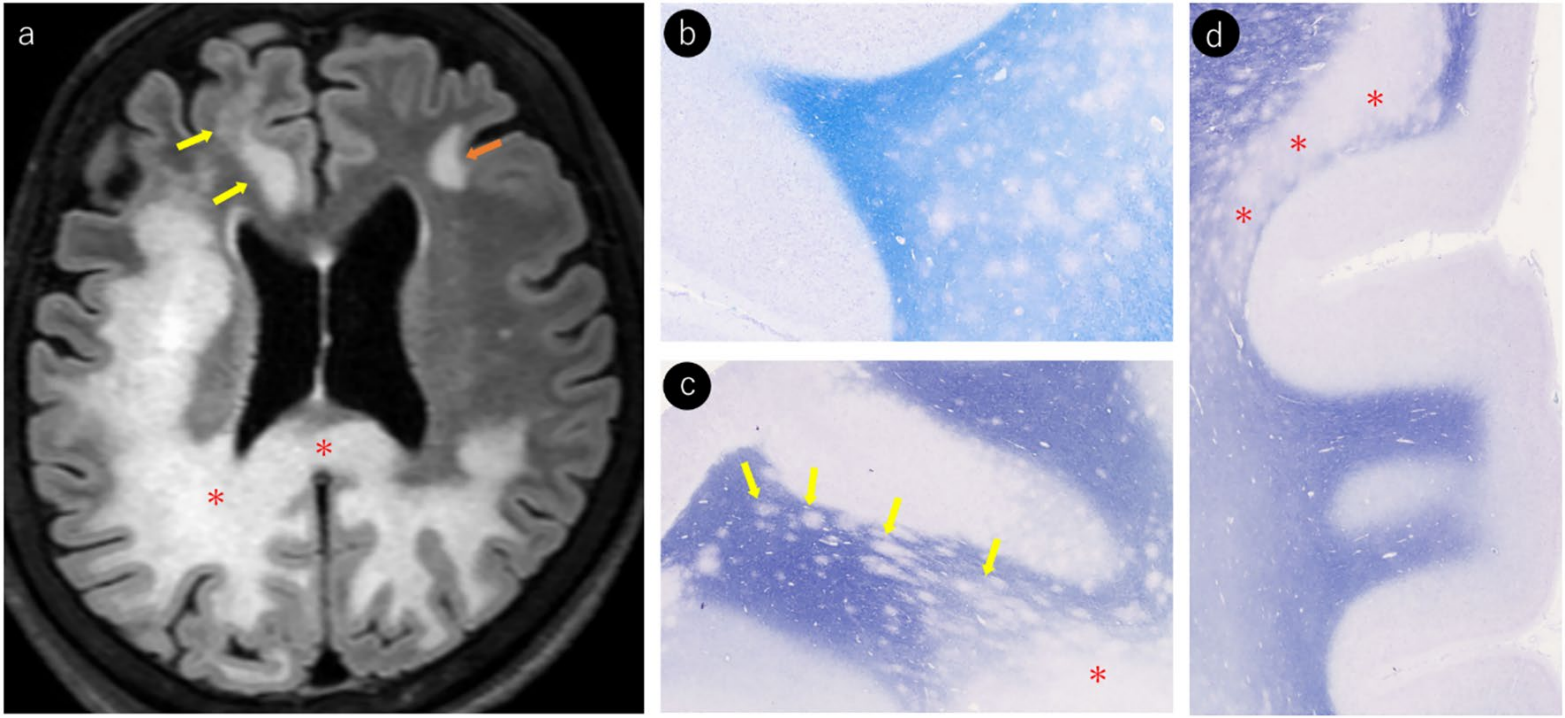
Klüver–Barrera (KB) staining of the autopsy brain from a woman in her 70 s with DLBCL and PML, along with MRI images taken 2 months prior. MRI and pathological findings (**a–d**): **a** FLAIR imaging demonstrates multifocal lesions throughout the brain, showing punctate lesions and subcortical white matter progression at the corticomedullary junction of the right frontal lobe (yellow arrows), lesions extending from the grey–white matter junction to the subcortical white matter in the left frontal lobe (orange arrow), and lesions traversing the corpus callosum with large confluent white matter lesions in the deep white matter of the right parietal lobe (*). Autopsy findings with KB staining (**b–d**): **b** multiple punctate lesions of varying sizes are observed in the subcortical to deep white matter. **c** Multiple discontinuous lesions extending along the axons in the subcortical regions were observed (yellow arrows). In deeper regions, the lesions coalesce to form large demyelinating plaques (*). **d** Linear lesions spread along the gray–white matter junction (*)

These three stages of pathological progression can be visualized as temporal changes on MRI scans. In addition, PML is characterized by the formation of multifocal brain lesions, and MRI can accurately depict the coexistence of lesions at various stages of progression (Fig. 11).

### Pathological early stage lesions and their MRI findings

According to Ono et al. [40], small demyelinating lesions are predominantly distributed around the juxtacortical/subcortical regions, with early stage lesion progression remaining locally confined to the subcortical white matter (Fig. 10). On MRI, these appear as linear and/or punctate T2WI/FLAIR hyperintense lesions along the corticomedullary junction (Figs. 1, 10) [51]. Over time, the lesions progressively spread to the subcortical white matter (Fig. 10). However, early stage demyelinating lesions can occur throughout the brain, including deep gray matter areas, such as the basal ganglia and thalamus, as well as the deep white matter. Early lesions in these regions often appear as punctate patterns on MRI (Fig. 6) [45].

### Local expansion and lesion progression

**Fig. 12:**
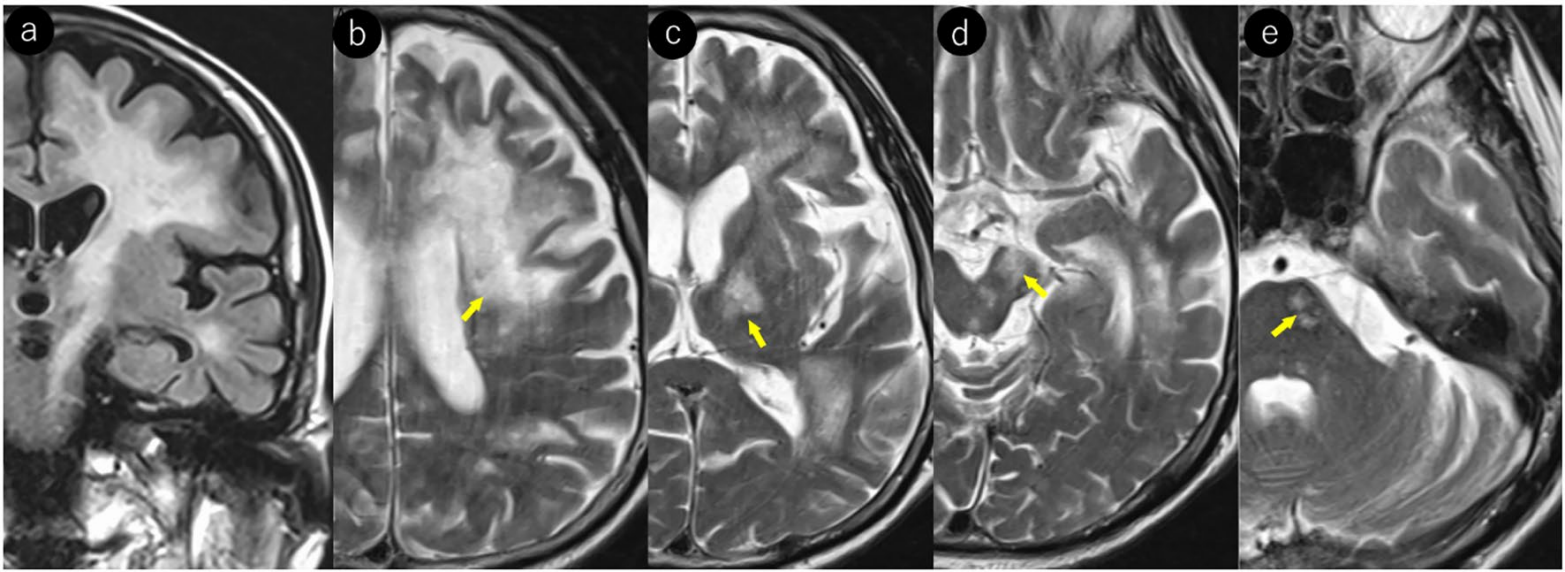
Man in his 40 s with PML in the setting of ALL presented with progressive right lower extremity paralysis. **a–e** Large lesion extending from the subcortical to the deep white matter of the left frontal lobe. The lesion shows caudal progression along the corticospinal tract (yellow arrow)

According to Ono et al. [40], initial lesions undergo both local expansion and progression to remote sites. Local expansion occurs as the replicated JCV spreads infection to adjacent cells while destroying host cells such as oligodendrocytes (Figs. 4, 6). Lesion progression occurs discontinuously along axons while preserving the axonal structure, potentially forming viral replication foci along nerve fibers and at distant sites. MRI characteristically demonstrates lesions extending along nerve fibers. When progression occurs along long nerve fibers, such as the corticopontine tract, corticospinal tract, corpus callosum, and transverse pontine fibers, it may present as “long lesions” [60, 61] [62] [63] (Fig. 12).

## Clinical subtypes of PML

PML presents with varying symptoms and imaging findings depending on the patient’s immune status and is generally classified into three types: “Classic PML,” “Inflammatory PML,” and “PML-IRIS” [1].

### Classic PML

Classic PML is the most frequently encountered disease with a severe clinical phenotype. It typically occurs in patients with severe immunodeficiency (e.g., HIV/AIDS patients not receiving antiretroviral therapy, patients with hematologic malignancies, such as leukemia and lymphoma) [1, 64, 65]. The disease generally follows a rapid course and has a poor prognosis. Without immune reconstitution, the lesions continue to expand over time, often leading to fatal outcomes. In HIV-infected patients, the median survival time for patients with PML before ART introduction was 6 months, with only 9% surviving beyond a year [26].

### Inflammatory PML

Inflammatory PML was first recognized as a distinct subtype in the mid-1990s with the introduction of ART [1, 66]. It is characterized by inflammatory responses against JCV due to the partial recovery of immune function.

**Fig. 13:**
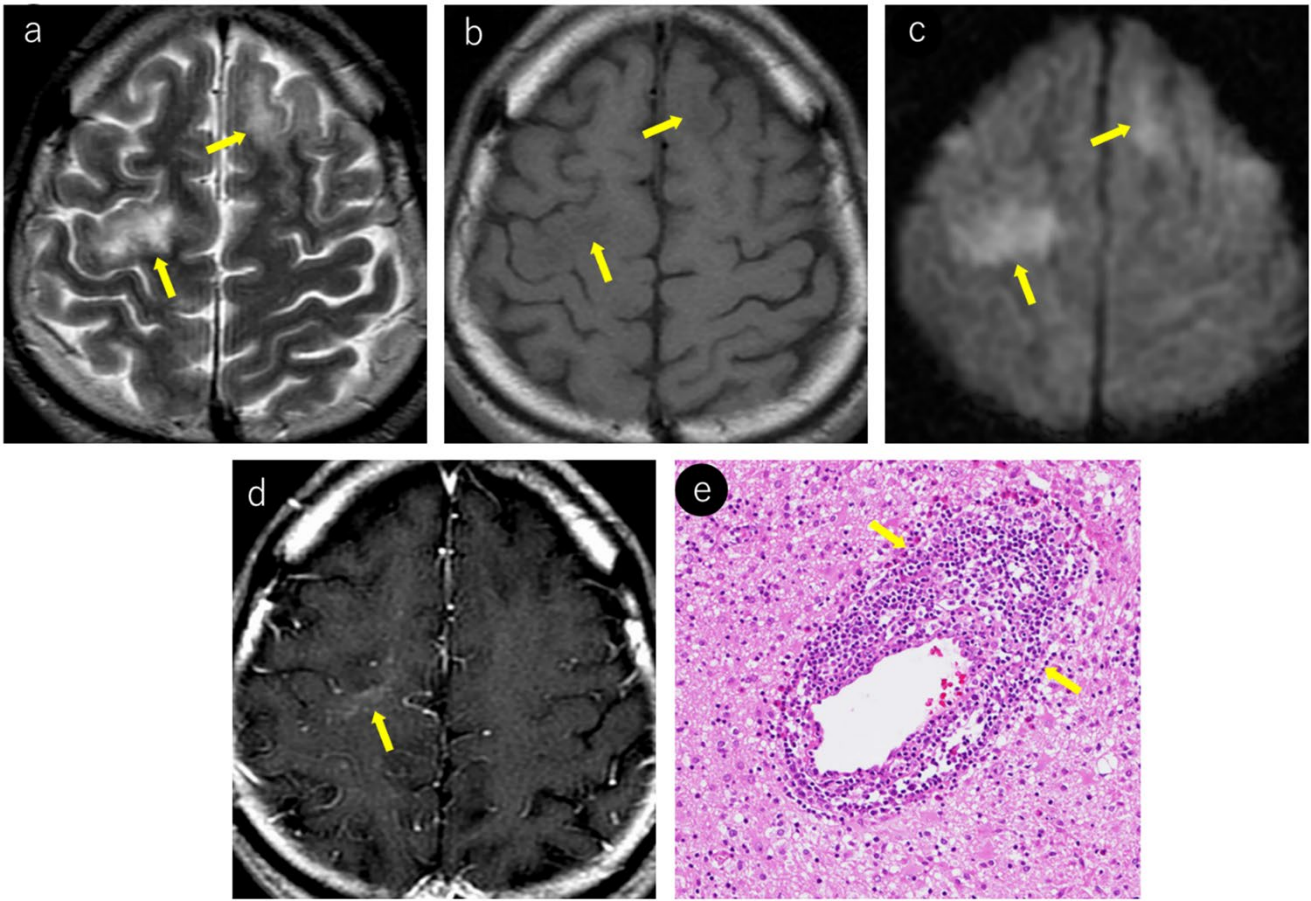
HIV-positive man in his 30 s developed progressive left-sided hemiparesis approximately 6 weeks after initiating antiretroviral therapy (ART). Initial MRI findings (**a–d**): **a** T2-weighted image showing heterogeneous hyperintense lesions in the subcortical white matter of both frontal lobes (yellow arrows). **b** These areas demonstrate subtle hypointensity on T1-weighted images (yellow arrows). **c** Lesions appear mildly hyperintense on diffusion-weighted imaging (yellow arrows). **d** Post-contrast T1-weighted image showing subtle punctate enhancement along the margin of the right frontal lobe lesion (yellow arrow). Despite negative CSF JCV test results, a brain biopsy of the right frontal lobe lesion confirmed the diagnosis of PML. **e** Pathological finding: hematoxylin and eosin (H&E) staining demonstrates inflammatory cell infiltration is observed in the perivascular spaces of the lesions (yellow arrows)

Recently, inflammatory PML has gained attention in patients undergoing natalizumab or fingolimod treatment [33, 67]. Pathologically, it shows intense perivascular lymphocytic inflammatory infiltration [68, 69]. On MRI, “patchy and punctate contrast enhancement” around PML lesions is considered an important indicator [45, 67] (Fig. 13). In natalizumab-associated PML, reports indicate that 41% of patients show contrast enhancement at the initial diagnosis [33]. This enhancement likely reflects partial immune reconstitution [1].

### PML IRIS

**Fig. 14:**
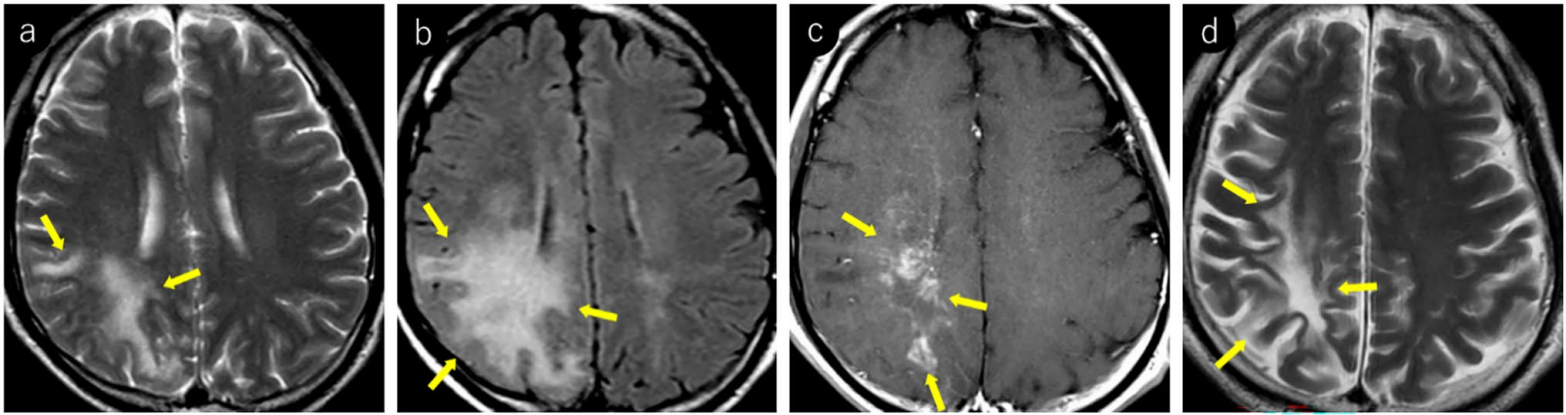
Previously untreated HIV-positive man in his 30 s presented with progressive visual impairment. **a** Initial MRI demonstrates T2 hyperintense lesions in the right occipitotemporal subcortical white matter (yellow arrows). CSF PCR confirms the diagnosis of PML. Two months after ART initiation: **b** FLAIR imaging showing the rapid expansion of existing PML lesions with mass effects and edematous changes (yellow arrows). **c** Post-contrast T1-weighted image showing patchy and punctate enhancement (yellow arrows). The combination of clinical and imaging findings led to the diagnosis of PML–IRIS. ART was continued, halting the progression of PML lesions. Long-term follow-up: **d** at the 10-year follow-up, the patient survived but showed marked brain atrophy in the affected regions (yellow arrows), with residual higher brain dysfunction and visual impairment

PML–IRIS is an extreme form of inflammatory PML and is characterized by rapid and intense immune responses [70]. Clinically, it presents with an acute worsening of neurological symptoms, while MRI findings include a mass effect, increased edema, midline shift, prominent contrast enhancement, and rapid expansion of the existing PML lesions [71–73] (Fig. 13). Areas of brain damage caused by PML–IRIS often result in atrophy and permanent sequelae [5] (Fig. 14).

Although inflammatory PML and PML–IRIS are favorable conditions for JCV elimination, steroid pulse therapy may be considered when excessive inflammation leads to severe neurological tissue damage. Various imaging techniques have been used to visualize active inflammation, including MRI spectroscopy [74], MRI arterial spin labeling [75, 76], and ^18^F-FDG–PET [77]. However, determining the severity of inflammatory PML or PML–IRIS based solely on imaging is challenging, and the ultimate judgment within the clinical context remains crucial [5].

## Other phenotypes of JCV infection

Although JCV infection has traditionally been understood primarily as PML affecting oligodendrocytes and astrocytes, atypical manifestations targeting various cells of the central nervous system have been reported. *Granular Cell Neuropathy (GCN)* is a newly recognized disease caused by direct JCV infection of cerebellar granule cells, first identified in HIV-associated PML and observed in various immunodeficient states [78, 79]. It presents as a cerebellar syndrome with progressive dysarthria and ataxia, with imaging showing progressive cerebellar atrophy [80, 81] (Fig. 7). In advanced stages of the disease, severe cerebellar atrophy and brainstem atrophy may be observed, along with the characteristic cruciform T2WI hyperintense signal in the brainstem known as the'hot cross bun sign'[79] (Fig. 7). *JCV encephalopathy* has been reported as a fulminant encephalopathy caused by the infection of pyramidal neurons in the cerebral cortex [82]. *JCV meningitis* has been reported as cases where the CSF tests positive for JCV without the classical PML lesions, presenting as meningitis or meningoencephalitis [83–85].

Remarkably, pathological examination reveals that PML patients frequently demonstrate concurrent JCV infections in multiple neural cell types beyond oligodendrocytes and astrocytes. Studies show high rates of co-infection in PML patients, with JCV infection found in cortical neurons (57% of cases [39]), cerebellar granule cells (79% of cases [86]), and leptomeningeal cells (29% of cases [87]). These findings suggest that JCV central nervous system infection represents a broader disease spectrum than traditionally recognized, with PML potentially being just one manifestation of a more diverse neurological syndrome [1].

## Asymptomatic and early stage PML, CSF-negative PML

### Clinical features

Traditionally, PML has been recognized as a condition with severe neurological symptoms at diagnosis and a poor prognosis. However, with the recent implementation of regular imaging screening for patients with MS receiving DMT, opportunities to detect PML in asymptomatic or early stages have increased [1]. Therefore, its early detection is clinically important.

There has been an increase in the number of reports of asymptomatic PML among patients using DMTs, particularly natalizumab. The survival rate of patients with PML discovered during the asymptomatic phase is notably higher (96%) than in symptomatic patients (75%) [4]. The risk factors for developing natalizumab-associated PML have been identified as: ①JCV antibody positivity, ②natalizumab treatment duration exceeding 2 years, and ③prior immunosuppressant use. Combining these factors enables the identification of populations with the highest risk. The highest risk group has an estimated incidence rate of 11.1 cases per 1,000 patients annually, while the lowest risk groups, such as those with negative antibodies, have an estimated incidence rate of less than 0.09 cases [18].

### Diagnostic challenges

#### CSF testing issues

A major challenge in early PML diagnosis is the detection sensitivity of JCV PCR testing in the CSF. In natalizumab-associated PML, JCV DNA levels in CSF are often very low, frequently below 100 copies/mL [30, 88], falling below the detection limits of standard commercial laboratories or quantitative test kits (100–200 copies/mL). Recently, low levels of CSF JCV have also been reported in PML cases associated with blood disorders and autoimmune diseases, and not just in those related to their treatment [2]. In early stage PML, the lesions are small, and smaller lesion volumes tend to correspond with lower JCV DNA levels [89] (Figs. 4, 5). In natalizumab-associated asymptomatic PML, lesions tend to be localized [1, 52]. In addition, in inflammatory PML, strong immune responses may suppress viral growth and transmission, significantly reducing JCV-infected cells, and potentially leading to negative CSF PCR results [35] (Fig. 12). Notably, reports indicate that up to 41% of natalizumab-associated PML cases present as inflammatory PML at the initial diagnosis [33], warranting particular attention.

### Test optimization and complementary approaches

A promising strategy for addressing these challenges is the implementation of ultrasensitive PCR testing, which has improved the detection limit to 10 or 20 copies/mL of CSF JCV [2, 21, 90]. When compared with the pathological diagnosis of PML through brain tissue examination (primarily brain biopsy), ultrasensitive PCR testing of CSF JCV (detection limit 20 copies/mL) demonstrated 85% sensitivity and 100% specificity, with sensitivity increasing to 95% when follow-up testing was included [90]. Furthermore, even more sensitive PCR testing (detection limit of 10 copies/mL) combined with ultrafiltration of viral particles in the CSF has proven useful in detecting extremely low levels of JCV in drug-associated PML [91, 92].

## MRI as an early diagnostic marker for PML

As previously discussed, PML’s clinical presentation and imaging findings of PML vary depending on immune status and underlying conditions. Careful detection and evaluation of these findings can increase the possibility of an early diagnosis of PML.

### ***MRI findings for early PML diagnosis (***Fig. 15***)***

**Fig. 15:**
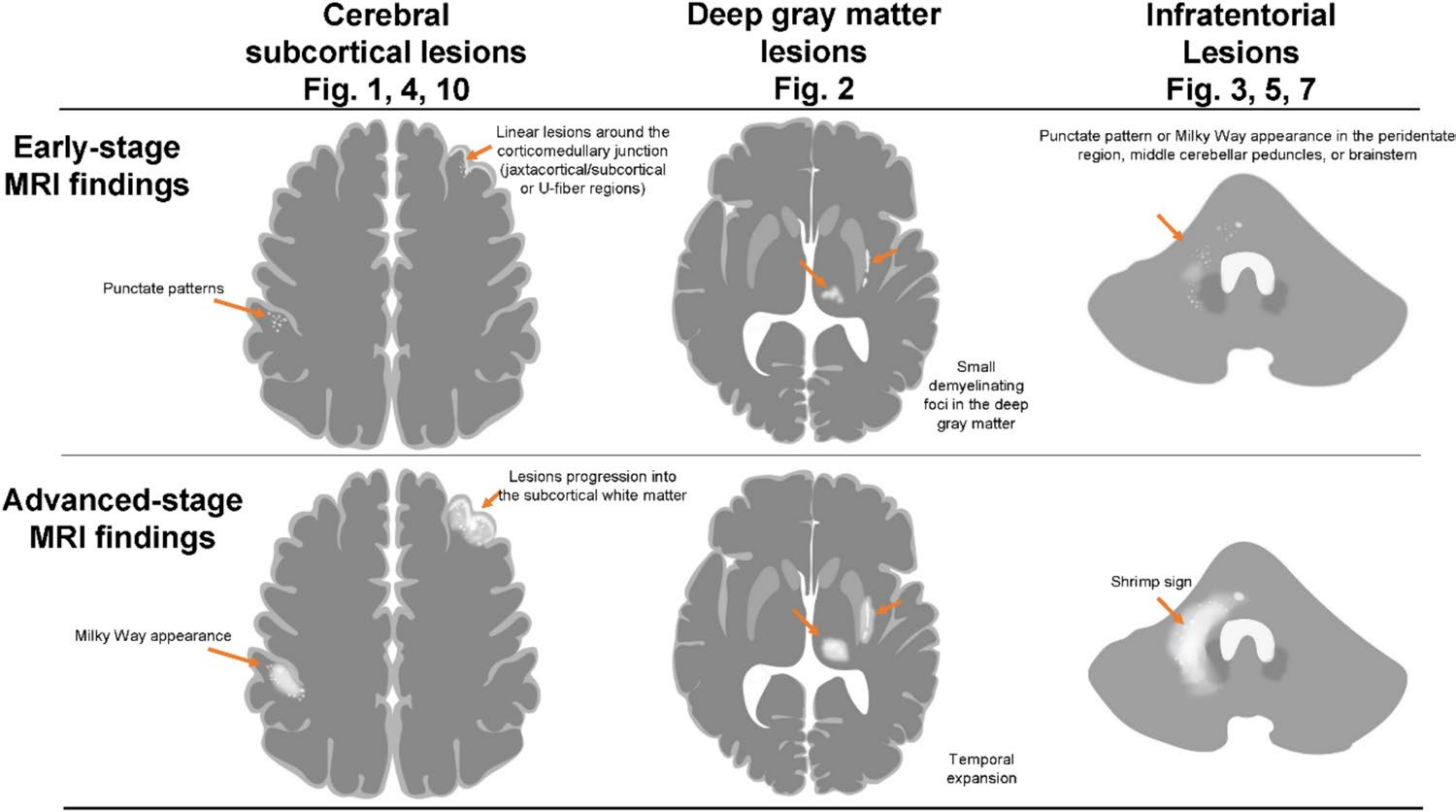
MRI findings for early PML diagnosis

#### Cerebral subcortical lesions (T2WI/FLAIR)

The subcortical region is the predilection site for PML. During the asymptomatic period or early stages, the identification of punctate patterns and a milky way appearance can be key diagnostic indicators. Linear lesions around the corticomedullary junction (jaxtacortical/subcortical or U-fiber regions) may be observed even in the early stages before the lesion progresses into the subcortical white matter. When SWI hypointensity is observed in the cortex or corticomedullary junction, careful attention should be paid to subtle T2WI/FLAIR hyperintensities in nearby gray–white-matter junctions and subcortical regions. Notably, asymptomatic and early stage PML lesions tend to be small and localized.

### Deep gray matter lesion (T2WI/FLAIR)

Deep gray matter lesions can form small demyelinating foci in the basal ganglia and thalamus. However, the absence of reduced ADC values, temporal expansion, and progression patterns along the nerve fibers are important distinguishing features suggestive of PML.

### Deep white matter lesion (T2WI/FLAIR)

A punctate pattern in the deep white matter may indicate early lesions. Lesion progression along nerve fibers such as the corpus callosum, corticospinal tract, corticopontine tract, and transverse pontine fibers is suggestive of PML. Progressive lesion expansion and fusion strongly indicated PML.

### Infratentorial lesion (T2WI/FLAIR)

In infratentorial lesions, predilection sites include the peridentate region, middle cerebellar peduncles, and brainstem. During the asymptomatic or early stages, attention should be paid to the punctate pattern and milky way appearance. The asymmetric “shrimp sign” is a highly diagnostic finding. Although rare, brainstem-limited PML exists, with lesion progression along nerve fibers being useful for differentiation [43].

### Signal intensity pattern evaluation (T1WI, DWI)

In evaluating these lesions, heterogeneous T1WI hypointensity (suggesting varying degrees of demyelination) and DWI hyperintensity (suggesting disease activity) are important findings when evaluating these lesions. However, early lesions, such as punctate patterns, localized milky way appearance, or linear lesions near the cortico-medullary junction, may not show DWI hyperintensity or T1WI hypointensity.

### Significance and characteristics of enhancement (post-contrast T1WI)

Contrast enhancement patterns vary according to the underlying disease and immune status. Patients undergoing partial immune reconstitution may exhibit patchy or punctate enhancement. Notably, natalizumab-associated asymptomatic PML often shows contrast enhancement at the initial presentation.

### Imaging follow-up strategy for early diagnosis

When PML is suspected at an early stage but CSF testing is negative, MRI monitoring for temporal changes should be performed [5]. If lesion expansion or progression along nerve fibers is observed over a short period (several weeks to months), PML should be reconsidered, and additional testing, such as ultrasensitive PCR testing for CSF JCV or brain biopsy, should be conducted [1, 32, 33, 89, 93–95].

### Future perspectives: new markers centered on SWI

The recently reported SWI findings may indicate early inflammatory changes in PML, and further research is required to validate their utility as predictive markers of early stage PML. When SWI hypointensity is observed in the cortex or corticomedullary junction, careful attention should be paid to subtle T2WI/FLAIR hyperintensities in nearby corticomedullary junctions and subcortical regions. Accumulation of knowledge regarding these findings may lead to improved PML screening and diagnostic accuracy. Prospective studies and large-scale cohort evaluations are required, particularly in high-risk patients.

## Conclusion

PML presents with various clinical phenotypes, including classic PML, inflammatory PML, and PML–IRIS, depending on the host immune status. Understanding and accurately identifying the characteristic MRI findings of PML often allows for high-precision diagnosis of PML based on imaging findings. Even when JCV is not detected on CSF testing, if imaging findings and clinical symptoms strongly suggest PML, additional testing such as ultrasensitive PCR testing for CSF JCV or brain biopsy should be considered.

## Ethical approval

This study was approved by our institutional ethical review board of Tokyo Metropolitan Cancer and Infectious Diseases Center Komagome Hospital (number 1659).
